# Entrapment of Probiotics in Water Extractable Arabinoxylan Gels: Rheological and Microstructural Characterization

**DOI:** 10.3390/molecules19033628

**Published:** 2014-03-24

**Authors:** Adriana Morales-Ortega, Elizabeth Carvajal-Millan, Francisco Brown-Bojorquez, Agustín Rascón-Chu, Patricia Torres-Chavez, Yolanda L. López-Franco, Jaime Lizardi-Mendoza, Ana L. Martínez-López, Alma C. Campa-Mada

**Affiliations:** 1 Research Center for Food and Development, CIAD, A.C. Carretera a La Victoria Km. 0.6, Hermosillo, Sonora 83304, Mexico; E-Mails: adriana.morales@estudiantes.ciad.mx (A.M.-O.); arascon@ciad.mx (A.R.-C.); lopezf@ciad.mx (Y.L.L.-F.); jalim@ciad.mx (J.L.-M.); ana.martinez@estudiantes.ciad.mx (A.L.M.-L.); acampa@ciad.mx (A.C.C.-M.); 2 University of Sonora, Rosales y Blvd. Luis D. Colosio, Col. Centro, Hermosillo, Sonora 83000, Mexico; E-Mails: fbrown@guaymas.uson.mx (F.B.-B.); pitorres@guayacan.uson.mx (P.T.-C.)

**Keywords:** polysaccharide gel, probiotic, prebiotic, cells entrapment, colon delivery

## Abstract

Due to their porous structure, aqueous environment and dietary fiber nature arabinoxylan (AX) gels could have potential applications for colon-specific therapeutic molecule delivery. In addition, prebiotic and health related effects of AX have been previously demonstrated. It has been also reported that cross-linked AX can be degraded by bacteria from the intestinal microbiota. However, AX gels have not been abundantly studied as carrier systems and there is no information available concerning their capability to entrap cells. In this regard, probiotic bacteria such as *Bifidobacterium longum* have been the focus of intense research activity lately. The objective of this research was to investigate the entrapment of probiotic *B. longum* in AX gels. AX solution at 2% (*w/v*) containing *B. longum* (1 × 10^7^ CFU/cm) formed gels induced by laccase as cross-linking agent. The entrapment of *B. longum* decreased gel elasticity from 31 to 23 Pa, probably by affecting the physical interactions taking place between WEAX chains. Images of AX gels containing *B. longum* viewed under a scanning electron microscope show the gel network with the bacterial cells entrapped inside. The microstructure of these gels resembles that of an imperfect honeycomb. The results suggest that AX gels can be potential candidates for the entrapment of probiotics.

## 1. Introduction

Biomaterials are used for encapsulation of probiotics and include natural and synthetic polymers [1]. The terms biocompatible and biodegradable are associated with many of these biomaterials because they are intended to be in contact with the digestive tract of the host. When choosing biomaterials it is important to know the physicochemical properties (chemical composition, morphology, mechanical strength, and stability in gastric and intestinal fluids) and to also meet the requirements of non-toxicity, resistance to gastric acidity and compatibility with respect to probiotic cells [2]. Arabinoxylans are polysaccharides constituted of a linear backbone of xylose units to which arabinose substituents are attached, some esterified with ferulic acid (FA) [3]. These polysaccharides have been classified as water extractable (WEAX) or water-unextractable (WUAX). WEAX can form covalent gels by oxidative coupling of FA resulting in the formation of dimers (di-FA) and trimers (tri-FA) of FA as covalent cross-linking structures [4]. Covalently cross-linked gels are generally strong, quickly formed, are not temperature dependent and exhibit no syneresis after long time storage. Furthermore, WEAX gels have interesting functional properties, which have not been exploited even though the neutral taste and odor of WEAX are desirable properties for industrial applications. WEAX networks have a high water absorption capacity (up to 100 g of water per gram of polymer) and they are not sensible to electrolytes or pH [5,6]. This property is essential for encapsulation of probiotics because the biomaterial must not dissolve in the stomach, but only into the gut [2]. WEAX gels could have potential applications for colon-specific therapeutic molecules delivery as biomaterials due to their porous structure, aqueous environment and dietary fiber nature [7]. In addition, prebiotic and health related effects of WEAX have been previously demonstrated [8]. It has been also reported that cross-linked WEAX can be degraded by bacteria from the intestinal microbiota [5,6]. In comparison to commonly studied polysaccharide gels (alginate, carrageenan or chitosan gels), WEAX gels have not been widely studied as carrier systems and to our knowledge there is no information available concerning their capability to entrap cells. In this regard, probiotic bacteria such as *Bifidobacterium longum* have been the focus of intense research activity in recent years. *B. longum* is found in the digestive tracts of infants, adults and elderly persons and its oxygen tolerance can be considered as a technological advantage for biomass production when compared with more strict anaerobic species such as *B. bifidum* and *B. adolescentis*. For these reasons, bifidobacteria are considered as more adequate probiotics for prevention and/or treatment of human intestinal disorders than lactobacilli [9]. The objective of this research was to investigate the entrapment of probiotic *B. longum* in WEAX gels and to study the rheological and microstructural characteristics of the gels formed.

## 2. Results and Discussion

### 2.1. Rheology

**Figure 1:**
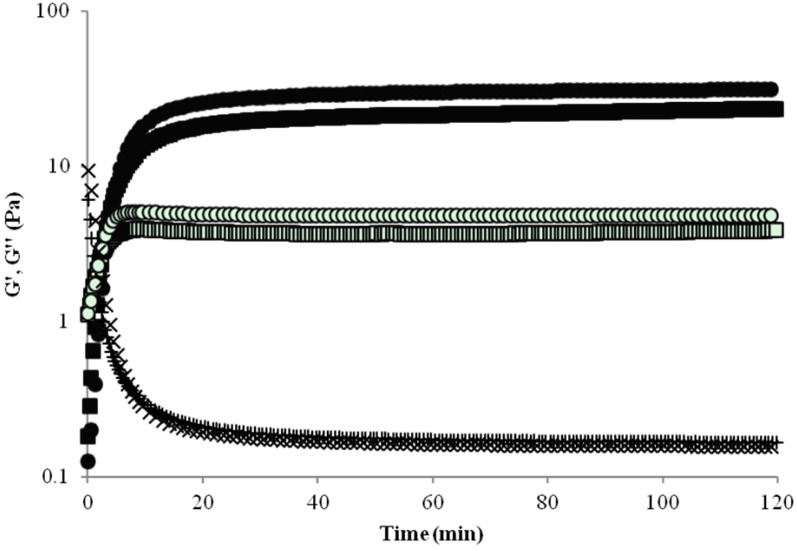
Gelation of 2% (*w/v*) WEAX solution, G' (○), G'' (●), tan δ (×) and 2% (*w/v*) WEAX solution containing *B. longum*, G' (■), G'' (□), tan δ (+). Laccase was used as cross-linking agent. Measurements at 25 °C, 1 Hz and 10% strain.

The kinetics of gelation of WEAX with and without *B. longum* was rheologically monitored by small amplitude oscillatory shear (Figure 1). In both cases the gelation process followed a characteristic profile with a rapid initial rise in storage (G') and loss (G'') moduli followed by a plateau region. This behavior reflects an initial formation of covalent linkages between FA of adjacent WEAX molecules producing a three dimensional network. Once sufficient cross-links have formed, movement of chains is impeded by the rigidity of the gel. The gelation time (tg), calculated from the crossover of the G' and G'' curves (G' > G'') was 4 min in both gels. The tg value indicates the sol/gel transition point and at this point G' = G'' or tan δ = G''/G' = 1 [10]. The final G' values of WEAX and WEAX containing *B. longum* were 31 and 23 Pa, respectively. The tan δ (G''/G') values calculated at 1Hz were from 0.160 and 0.165 in WEAX gels with and without bacteria, respectively, indicating the presence of an elastic covalent system [11]. The slight increase in tan δ values in WEAX gels containing *B. longum* indicated a higher viscous contribution to the network structure which could be related to an increase in the polymer chain flexibility in the gel [10]. The entrapment of *B. longum* decreased gel elasticity probably by affecting the physical interactions taking place between WEAX chains. The addition of *B. longum* to WEAX led, after 2 h of laccase action, to a 26% loss in G'. This decrease in G' value could be related to a reduction in physical interactions between polysaccharide chains, which would affect network connectivity. This is consistent with the slight increase in tan δ values in the presence of *B. longum.*

**Figure 2:**
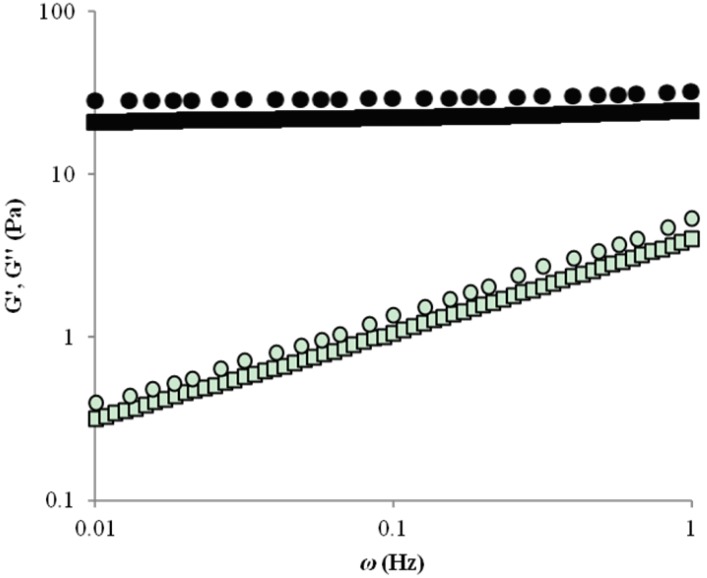
Mechanical spectra of 2% (*w/v*) WEAX gel, G' (●), G'' (○) and 2% (*w/v*) WEAX gel containing *B. longum*, G' (■), G'' (□). Measurements at 25 °C and 10% strain.

The mechanical spectra of WEAX after 2 h gelation (Figure 2), was typical of solid-like materials with a linear G' independent of frequency and G'' smaller than G' and dependent of frequency. For both gels obtained G'' increased with frequency, indicating WEAX chains mobility in the gel [11]. Nevertheless, G'' values were almost superposed indicating that the viscous contribution to the gel structure was similar. These mechanical spectra typical of a gel have been previously reported for WEAX gels [4,6] but, to our knowledge the present study represent the first report on microorganisms entrapment in WEAX gels.

### 2.2. Microstructure

**Figure 3:**
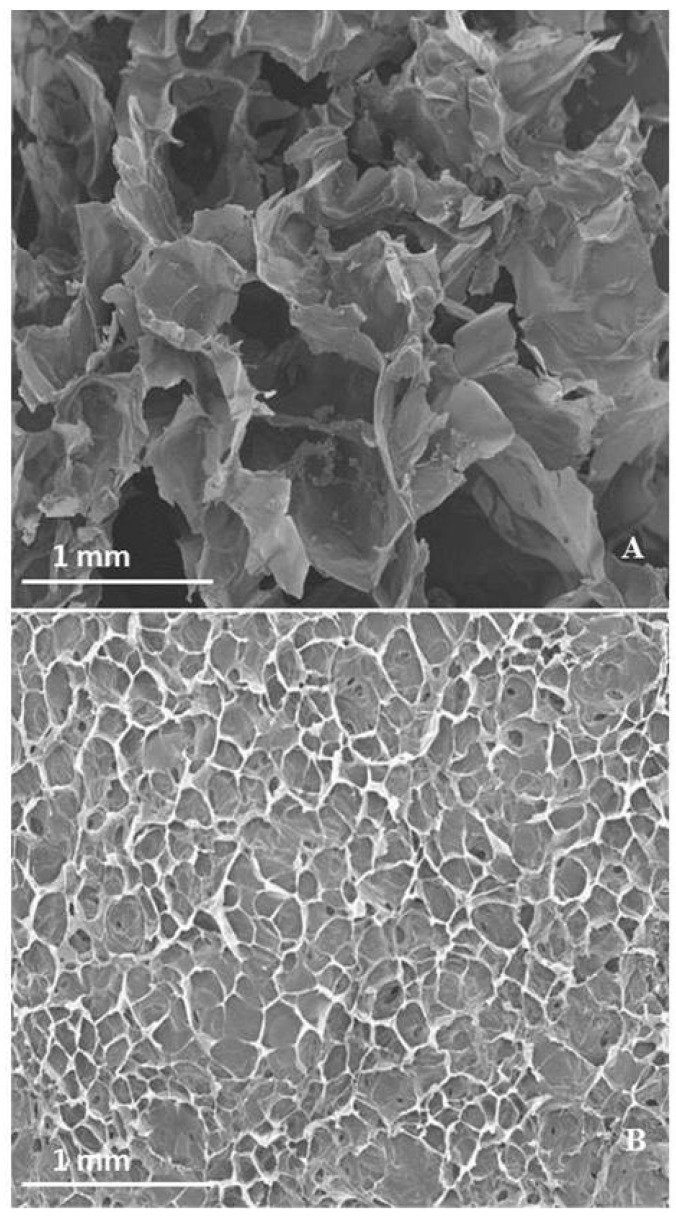
SEM micrographs of lyophilized WEAX gel (**A**) and WEAX gel containing *B. longum* (**B**) at 35× magnification.

The microstructural characteristics of lyophilized WEAX gel and WEAX gel containing *B. longum* was observed by scanning electron microscopy (SEM). As reported in Figure 3A,B, WEAX gel and WEAX gel containing *B. longum* present a microstructure resembling an irregular honeycomb structure. This morphological microstructure is similar to that reported before for lyophilized wheat and maize AX gels [12,13,14,15]. WEAX gel and WEAX gel containing *B. longum* present average inner cell dimensions of approximately 200 × 400 (Figure 3A) and 100 × 200 µm (Figure 3B). The lower cell dimensions in WEAX gel containing *B. longum* indicate a better preserved structure in this lyophilized polymeric material. It is known that an important aspect of achieving high quality frozen material, particularly with a high water content such as gels, is the size of ice crystals formation. In general, finer ice crystals result in a better preserved structure [16]. At subzero temperatures small amounts of liquid water continue to growth into big crystals, but the presence of antifreeze agents may modify this crystal growth. The presence of antifreeze proteins has been reported in several bacteria species, such as *Pseudomonas fluorescens* and *Marinomonas primoryensis*. In addition, some bacteria can secret extracellular polymeric substances presenting ice recrystallization inhibition and low temperature protection. For example, *Xanthomonas campestris* secrets xanthan gum which shows ice recrystallization inhibition [17]. To our knowledge, neither antifreeze activity nor extracellular polymeric substances presenting ice recrystallization inhibition have been investigated for *B. longum*. In this regard, the mechanism of WEAX gel structure preservation by *B. longum* should be further studied.

**Figure 4:**
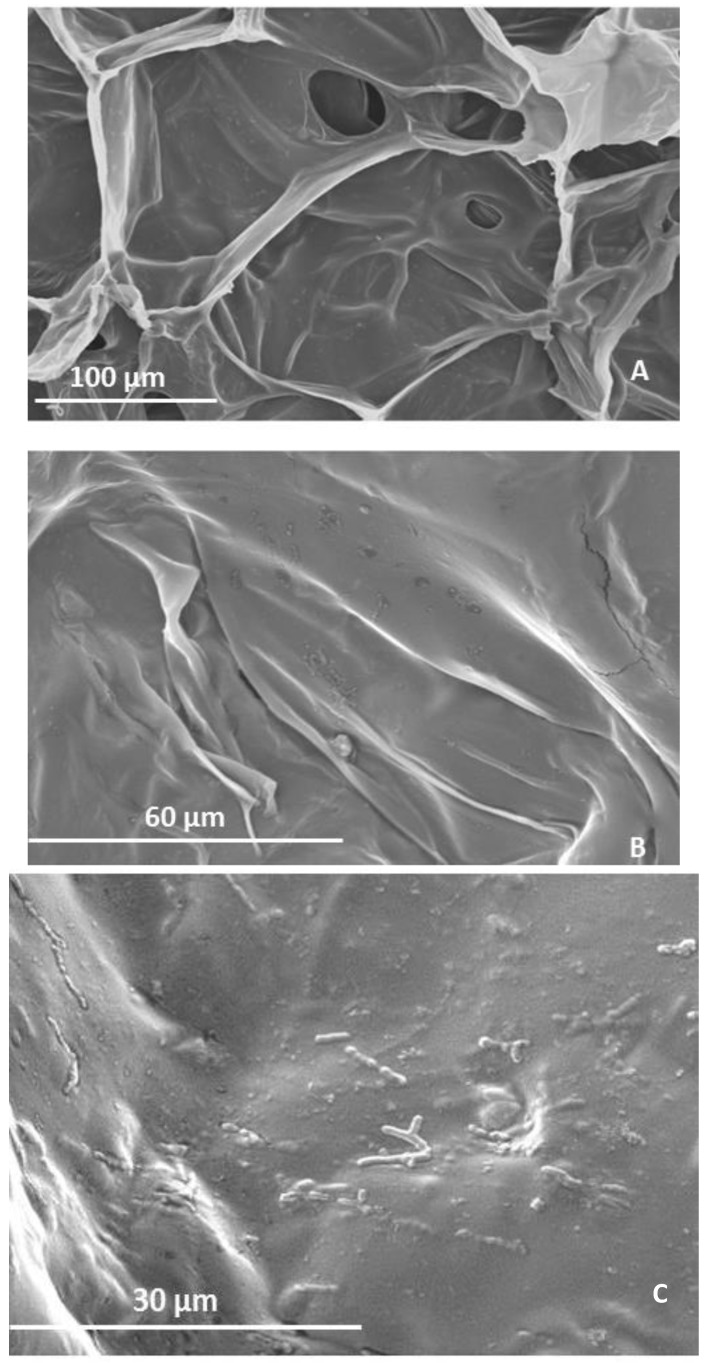
SEM micrographs of lyophilized WEAX containing *B. longum* gel at 350× magnification (**A**), 1,000× magnification (**B**) and 2,000× magnification (**C**).

Scanning electron micrographs in Figure 4 provide evidence that bacteria were entrapped within the walls of the gel structure, showing a uniform distribution. These results are not in agreement with the findings of other reports, where calcium-alginate microparticles were formed and images obtained by cryo-SEM. They observed bacteria aggregates within the microparticles, with void space around the bacteria suggesting that the presence of bacteria disrupted gelation of alginate in their immediate vicinity [18]. Additionally, SEM images of microspheres using a mixture of alginate with prebiotic (sugarbeet and chicory) and probiotic (*Staphylococcus succinus* and *Enterococcus fecium*) showed the bacteria immobilized in the matrix presenting a randomly distribution and the surfaces of the beads were found to be rough [19]. In this study, SEM revealed that the presence of bacteria does not interfere with the gelation of AX, but indicates a better preserved structure in the lyophilized gel with *B. longum*. Moreover, SEM images of *Lactobacillus plantarum* entrapped in a matrix of κ-carrageenan with protein and varying the weight ratios, formed spherical microparticles with a smooth surface, forming an interconnected matrix with *L. plantarum* bound to it. As the weight ratio increased, larger and more spherical particles were grouped into larger clusters. Those structural features were reflected in the rheological properties resulting in lower G’ and G’’ values. Similar results were observed in our study, since the entrapment of *B. longum* decreased gel elasticity probably by affecting the physical interactions taking place between WEAX chains [20]. Furthermore, Figure 4C shows the double head of the *B. longum*. To our knowledge, studies reporting microphotographs of probiotics entrapped in polymer networks are not common and there is no information available concerning WEAX gels capability to entrap cells.

## 3. Experimental

### 3.1. Materials

WEAX were extracted from the endosperm of a spring wheat variety (Tacupeto F2001). WEAX presented an A/X ratio of 0.66, a [η] of 3.5 dL/g, and Mv of 504 kDa [21]. The FA content of WEAX was 0.526 μg/mg WEAX. Bacteria *Bifidobacterium longum* ATCC 15708 and a commercial laccase (benzenediol:oxygen oxidoreductase, E.C.1.10.3.2) from *Trametes versicolor* were used. All chemical reagents were purchased from Sigma Chemical Co. (St Louis, MO, USA). The grain sample was milled by using Quadrumat Sr mill (Brabender, South Hackensack, NJ, USA) according to the AACC method 26–10 [22].

### 3.2. WEAX and WEAX/B. Longum Gelation

A WEAX solution (2% *w/v*) and a WEAX solution (2% *w/v*) containing *Bifidobacterium longum* (1 × 10^7^ CFU/cm) were prepared in 0.05 M citrate phosphate buffer pH 5.5. Laccase (1.675 nkat per mg WEAX) was used as cross-linking agent. Gels were allowed to set for 2 h at 25 °C [4].

### 3.3. Rheology

Small amplitude oscillatory shear was used to follow the gelation process of WEAX and WEAX/*B. longum*. Cold (4 °C) WEAX solution (2% *w/v*) or WEAX solution (2% *w/v*) containing *B. longum* (1 × 10^7^ CFU/cm) prepared in 0.05 M citrate phosphate buffer pH 5.5 were mixed with laccase and immediately poured on plate-plate geometry (4.0 cm in diameter) of a strain controlled rheometer (Discovery HR-3 rheometer, TA Instruments, New Castle, DE, USA). Exposed edges were covered with silicone oil to prevent evaporation. WEAX and WEAX/*B. longum* gelation was started and monitored at 25 °C for 2 h by recording the storage (G') and loss (G'') moduli. Measurements were carried out at 1.0 Hz frequency and 10% strain. The mechanical spectra of gels were obtained by frequency sweep from 0.01 to 10.0 Hz with a 10% strain at 25 °C [4,23].

### 3.4. Microstructure

WEAX gels and WEAX/*B. longum* gels were frozen at −20 °C and lyophilized at −37 °C/0.133 mbar overnight in a Freezone 6 freeze drier (Labconco, Kansas, MO, USA). The internal structure of freeze-dried WEAX gel was studied by scanning electron microscopy (JEOL 5410LV, JEOL, Peabody, MA, USA) at low voltage (20 kV). SEM image was obtained in Secondary Electron Imaging (SEI) mode.

## 4. Conclusions

In this study, it was possible to entrap *B. longum* in WEAX gels for the first time. The presence of bacteria results in a slight reduction of AX gel elasticity, probably by affecting the physical interactions taking place between WEAX chains. Images of WEAX gels containing *B. longum* viewed under scanning electron microscopy show the gel network with the bacterial cells entrapped inside. The microstructure of these gels resembles an imperfect honeycomb. The results suggest that WEAX gels can be potential candidates for the entrapment of probiotics for subsequent synbiotic matrix design. Further research would be needed to determine the stability of these matrices in an acidic medium, and their disintegration or dissolution simulating the digestive tract environment.
